# Using a summary measure for multiple quality indicators in primary care: the Summary QUality InDex (SQUID)

**DOI:** 10.1186/1748-5908-2-11

**Published:** 2007-04-02

**Authors:** Paul J Nietert, Andrea M Wessell, Ruth G Jenkins, Chris Feifer, Lynne S Nemeth, Steven M Ornstein

**Affiliations:** 1 Department of Biostatistics, Bioinformatics, and Epidemiology, Medical University of South Carolina, Charleston, SC (USA); 2 Department of Pharmacy and Clinical Sciences, South Carolina College of Pharmacy, Medical University of South Carolina campus, Charleston, SC (USA); 3 Department of Family Medicine, Medical University of South Carolina, Charleston, SC (USA); 4 Department of Family Medicine, Keck School of Medicine, University of Southern California, Los Angeles, CA (USA); 5 College of Nursing and Clinical Services, Medical University of South Carolina, Charleston, SC (USA)

## Abstract

**Background:**

Assessing the quality of primary care is becoming a priority in national healthcare agendas. Audit and feedback on healthcare quality performance indicators can help improve the quality of care provided. In some instances, fewer numbers of more comprehensive indicators may be preferable. This paper describes the use of the Summary Quality Index (SQUID) in tracking quality of care among patients and primary care practices that use an electronic medical record (EMR). All practices are part of the Practice Partner Research Network, representing over 100 ambulatory care practices throughout the United States.

**Methods:**

The SQUID is comprised of 36 process and outcome measures, all of which are obtained from the EMR. This paper describes algorithms for the SQUID calculations, various statistical properties, and use of the SQUID within the context of a multi-practice quality improvement (QI) project.

**Results:**

At any given time point, the patient-level SQUID reflects the proportion of recommended care received, while the practice-level SQUID reflects the average proportion of recommended care received by that practice's patients. Using quarterly reports, practice- and patient-level SQUIDs are provided routinely to practices within the network. The SQUID is responsive, exhibiting highly significant (p < 0.0001) increases during a major QI initiative, and its internal consistency is excellent (Cronbach's alpha = 0.93). Feedback from physicians has been extremely positive, providing a high degree of face validity.

**Conclusion:**

The SQUID algorithm is feasible and straightforward, and provides a useful QI tool. Its statistical properties and clear interpretation make it appealing to providers, health plans, and researchers.

## Background

Assessment of the quality of primary care is becoming a clear priority in national healthcare agendas. To evaluate the care provided to patients with chronic illnesses and clinical conditions that affect large segments of the population, numerous quality indicators and performance measures have been developed. For example, performance measurements by the US Centers for Medicare and Medicaid Services (CMS) Physician Focused Quality Initiative, including the Doctor's Office Quality Project, Doctor's Office Quality Information Technology Project, and Vista-Office Electronic Health Record, are being implemented nationally to assess the care of Medicare beneficiaries, support clinicians in providing appropriate treatment, prevent avoidable health problems, and evaluate the concept of pay-for-performance [1]. Other examples include performance measures endorsed by the US National Committee for Quality Assurance, the National Quality Forum, the American Medical Association/Physician Consortium for Performance Improvement, and the Ambulatory Care Quality Alliance [1].

Implementation of research into clinical practice has been facilitated through multiple QI strategies, including audit and feedback [2,3]. Providing feedback to clinicians on their performance related to specific indicators is one of the components used to improve the quality of care provided. In situations such as this, where numerous quality indicators are utilized, it has been argued that there may be instances in which fewer numbers of more comprehensive indicators are preferable [4]. For example, during quality improvement (QI) projects involving multiple process and/or outcome measures within multiple clinical domains, efforts to improve quality in one area may yield a decline in quality in another area. In such circumstances, a summary measure may provide clinicians and researchers with a better sense of whether their efforts (or lack thereof) result in net increases or decreases in quality.

Several earlier publications have discussed algorithms used to summarize quality measures in different arenas of the healthcare system. For example, CMS has developed a system for summarizing quality indicators for hospitals [5], and investigators with RAND Corporation have created a mechanism for assessing overall quality of care provided to various communities around the US [6,7]. The US Department of Veterans Affairs (VA) has developed similar evidence-based measures, incorporating a "prevention index" and a "chronic disease index" as a means of encouraging better provider performance [8]. Likewise, several papers have addressed statistical methodology (e.g., latent variable models [9], factor analysis [4], and Bayesian hierarchical regression models [10]) for physician, hospital, or health plan 'profiling,' in which an index is created that compares the overall quality of care provided among various physicians. Global statistical tests have also been proposed for comparisons of multiple correlated outcomes, typically used within the clinical trials setting; however, their use in composite quality indices has been minimal [11-14]. Although generally such sophisticated statistical methods provide summaries across multiple quality domains and account for correlation among the individual measures of quality, with the exception of the CMS, RAND, and VA methodologies, the composite indices proposed in those papers do not have a direct clinical interpretation. Additionally, these methods may be inadequate when the composite score includes individual indicators that are not applicable to selected groups of patients.

This paper outlines the construction, validation, and use of the Summary Quality Index (SQUID), a composite measure summarizing the quality of care provided by primary care providers. It was developed in the Practice Partner Research Network (PPRNet), a practice-based research network, for use in a QI demonstration project. PPRNet is a network of ambulatory primary care clinicians throughout the US who use a common electronic medical record (Practice Partner, Seattle, WA). Data from outpatient encounters (e.g., demographics, diagnoses, medications, laboratory results, and vital signs) are remitted quarterly to PPRNet staff at the Medical University of South Carolina, where the data are prepared for analysis and summarized in practice performance reports. Throughout this process, only active adult patients over 18 years old are included. Within PPRNet, a patient is considered active at any point in time if he/she has had a progress note recorded in the electronic medical record in the prior 12 months; a patient is considered to have an active medication if it was prescribed in the prior 12 months. As of the third quarter 2005, 89 practices were represented. Although the SQUID has been developed within the PPRNet setting, the algorithm used to create it is generalizable to many other healthcare settings.

As a part of the QI demonstration project entitled Accelerating the Translation of Research into Practice (A-TRIP), an intervention which spanned 42 months (January 2003 through June 2006), this group of PPRNet clinicians has been provided with quarterly reports on 36 unique quality indicators (see Table 1). Thirty-one of these indicators are process measures, while five are outcome measures. As is customary with performance measurement [15], the indicators were chosen based on the ability of providers to act on them, supporting evidence and national prevention and disease management guidelines [16-28], and availability of data from the EMR. Chosen indicators are in the following domains: prevention and management of hypertension (HTN), coronary heart disease (CHD), stroke, diabetes mellitus (DM), and respiratory/infectious disease, cancer screening, immunizations, substance abuse and mental health, nutrition and obesity, and inappropriate prescribing in elderly patients. The A-TRIP QI demonstration project was comprised of three specific types of interventions: practice performance reports (audit and feedback), optional semi-annual site visits to practices for academic detailing and participatory planning, and optional annual network meetings to share 'best practice' approaches. The logic and supporting theory of the A-TRIP intervention has been published elsewhere [29]. The purpose of this paper is to summarize the development of a statistically robust and clinically meaningful composite summary measure that would help the research team and individual practices evaluate the overall progress of a QI demonstration project.

**Table 1:** A-TRIP quality indicators and eligibility criteria

Quality indicator	Eligibility criteria	Target
*Process Measures*		
BP monitoring	All adults	Within past 6 months (DM or HTN); otherwise within past 2 years
Total cholesterol measurement	All adults	Within past 5 years
HDL measurement	All adults	Within past year (DM); otherwise within past 5 years
LDL measurement	DM, CHD, or other atherosclerotic disease	Within past year
Triglyceride measurement	DM	Within past year
HgbA1C measurement	DM	Within past 6 months
Pap test	Women without hysterectomy who are > = 18 years old but 65 years old	Within past 3 years
FOBC, Sigmoidoscopy, or Colonoscopy	Age > = 50	FOBC within past year, or sigmoidoscopy within past 5 years, or colonoscopy within past 10 years
Mammogram	Women > = 40 years old	Within past 2 years
Td vaccine	Age > = 12	Within past 10 years
Flu vaccine	Age > = 65 or [Age > = 18 and (DM, Asthma, COPD, CHD, HF, renal disease, or alcohol abuse)]	Within past year
Pneumococcal vaccine	Age > = 65 or [Age > = 18 and (DM, Asthma, COPD, CHD, HF, renal disease, or alcohol abuse)], different frequencies	Ever
Hep A vaccines (n> = 2)	Liver disease	Ever
Chlamydia screening	Women 16–25 years old	Within past year
Depression screening	Age > = 18	Within past 2 years
Alcohol screening	Age > = 18	Within past 2 years
Alcohol counseling	Alcohol abusers	Within past year
Tobacco counseling	Smokers	Within past year
Diagnosing HTN	Adults with 3 BPs > 140/90 mmHg in past year	Diagnosis of HTN
Blood glucose test	Obesity	Within past year
Diet/nutritional counseling	Obesity, HTN, hyperlipidemia, or DM	Within past year
ACE or ARB	(DM and HTN) or HF	Active ACE or ARB Rx
Lipid lowering Rx	CHD or other ASD	Active lipid lowering Rx
Beta blocker	HF	Active beta blocker Rx
Anti-thrombotic agent	AF or (> 40 years old and one or more of the following: HTN, hyperlipidemia, DM, other atherosclerotic disease)	Active aspirin or warfarin Rx (AF patients); otherwise active aspirin Rx
Anti-inflammatory agent	Asthma	Active anti-inflammatory agent Rx
Anti-depressant	Depression	Active anti-depressant Rx
Urinary microalbumin	DM	Within past year
*Prescriptions with contraindications*		
Antibiotic agents	URI, pharyngitis, or bronchitis in past month	Antibiotic Rx within 3 days of visit for URI, pharyngitis, or bronchitis
Use of any drug that's always inappropriate	Age > = 65	Active always inappropriate Rx
Use of any drug that's rarely appropriate	Age > = 65	Active rarely appropriate Rx
*Outcome Measures*		
BP control	DM, HTN	< 130/80 mmHg (DM) or < 140/90 mmHg (HTN)
HDL control	DM	> 45 mg/Dl
LDL control	DM, CHD, or other atherosclerotic disease	< 100 mg/Dl
Triglyceride control	DM	< 150 mg/Dl
HgbA_1_C control	DM	< 7%

## Methods

The algorithm for creating the composite quality measure was developed during the A-TRIP project, which was approved by the Institutional Review Board of the Medical University of South Carolina. The algorithm for creating the SQUID from the 36 quality measures includes 1) determining which patients are eligible for which process and outcome measures; 2) determining which patients have met their desired clinical targets; and 3) calculating SQUIDs for each patient and for each practice.

### Determining which patients are eligible for which process and outcome measures

The first step in the SQUID algorithm involves counting the number of process and outcome measures for which the patient is eligible. For example, only patients with DM are eligible for hemoglobin A_1c _(A1C) monitoring. An indicator variable is thus created, with a one indicating that a given patient is eligible (i.e., has DM) for the particular measure of interest (i.e., A1C monitoring), and a zero indicating that the patient is not eligible (i.e., does not have DM). These indicator variables are denoted by E_i_, where E_1 _is an indicator variable reflecting eligibility for the first unique measure, E_2 _is an indicator variable reflecting eligibility for the second unique measure, etc., and where 'i' ranges from one to thirty-six, the total number of unique process and outcome measures. The total number of measures for which a patient is eligible is thus E = ∑i=136Ei
 MathType@MTEF@5@5@+=feaafiart1ev1aaatCvAUfKttLearuWrP9MDH5MBPbIqV92AaeXatLxBI9gBaebbnrfifHhDYfgasaacH8akY=wiFfYdH8Gipec8Eeeu0xXdbba9frFj0=OqFfea0dXdd9vqai=hGuQ8kuc9pgc9s8qqaq=dirpe0xb9q8qiLsFr0=vr0=vr0dc8meaabaqaciaacaGaaeqabaqabeGadaaakeaadaaeWbqaaiabdweafnaaBaaaleaacqWGPbqAaeqaaaqaaiabdMgaPjabg2da9iabigdaXaqaaiabiodaZiabiAda2aqdcqGHris5aaaa@36BC@. Note that patients with greater numbers of diseases/medical conditions will be eligible for more process and outcome measures, and thus the total (E) may be used subsequently in analyses that need to adjust for the level of patient complexity. Also, all adult patients over 18 years old are eligible for at least six process measures, including blood pressure (BP), total cholesterol and high density lipoprotein (HDL) cholesterol monitoring, tetanus/diphtheria vaccine, depression, and alcohol screening.

### Determining which patients have met their desired targets

The next set of indicator variables reflects whether or not the patient has met the targets for the eligible quality measures. For process measures, the target has been met if the process has been performed within some pre-specified time frame (e.g., past six months, past year). For outcome measures, the target has been met if the measure of interest is under (in the case of BP, low density lipoprotein [LDL], triglycerides, and A1C) or over (for HDL) the guideline recommendation. These targets may vary according to the patients' co-morbidities. For example, the BP control target is less than 140/90 mmHg for patients with HTN and less than 130/80 mmHg for patients with DM. Patients with both HTN and DM need to meet the more stringent target (i.e., less than 130/80 mmHg). The relevant indicator variables (M_j_'s) are then summed so that M, the total number of process/outcome targets that a patient has met, is defined as M = ∑j=1EMj.
 MathType@MTEF@5@5@+=feaafiart1ev1aaatCvAUfKttLearuWrP9MDH5MBPbIqV92AaeXatLxBI9gBaebbnrfifHhDYfgasaacH8akY=wiFfYdH8Gipec8Eeeu0xXdbba9frFj0=OqFfea0dXdd9vqai=hGuQ8kuc9pgc9s8qqaq=dirpe0xb9q8qiLsFr0=vr0=vr0dc8meaabaqaciaacaGaaeqabaqabeGadaaakeaadaaeWbqaaiabd2eannaaBaaaleaacqWGQbGAaeqaaaqaaiabdQgaQjabg2da9iabigdaXaqaaiabdweafbqdcqGHris5aOGaeiOla4caaa@36E3@.

### Calculating SQUIDs at the patient and practice level

Once E and M have been determined for each patient, the patient-level SQUID is simply calculated by dividing M (measures met) by E (eligible measures), thus reflecting the proportion of relevant targets achieved for that patient. Because the SQUID is a proportion, it ranges from 0.0% to 100.0%. Note that the SQUID incorporates both individual process and outcome indicators, as has been done for specific clinical domains in other studies [30,31].

Another feature of the SQUID is that it can be calculated at the patient, provider, or practice-level. The practice-level SQUID is calculated as the average of all the patient-level SQUIDs among active patients in the practice. The practice-level SQUID thus reflects the average proportion of relevant targets achieved for patients in the practice. In A-TRIP, provider-level SQUIDs were not reported; however, these could easily be calculated in other settings.

### Use of the SQUID in QI

Once the patient-level and practice-level SQUIDs were developed, they were incorporated into practice quality performance reports provided to A-TRIP practices on a quarterly basis. From January 2003 to April 1, 2005, participating A-TRIP practices received quarterly performance reports that only encompassed performance on the individual quality measures. After April 1, 2005, practice reports included a statistical process control chart that summarized the practices' performance on their practice-level SQUID. These charts, similar to ones used for the individual quality measures, mapped the practices' SQUID scores on a monthly basis over the past 24 months. Practices were provided these reports as part of the A-TRIP project through the end (i.e., June 2006) of the QI project, and final analyses of the A-TRIP project included an assessment of the change in practice-level SQUID scores over the 3.5 year study time frame. The analysis of the change in SQUID scores during A-TRIP was also presented to providers during the 2005 and 2006 A-TRIP network meetings, which were designed to help providers improve quality by listening to 'best practice' approaches and by discussing their ideas with one another. In fact, the 'best' practices were determined, in part, by performance on their SQUID scores.

In addition to the practice-level reports, throughout the A-TRIP QI effort practices have been provided with patient-level reports, similar to a patient registry. These reports consist of Excel spreadsheets with embedded filters and macros that can help the practice identify their patients not at goal on individual quality measures. Starting with the 2^nd ^quarter 2005 practice-level reports, the patient-level SQUID was added to these reports. By having this overall quality score calculated for individual patients, practices were then able to identify, for example, their patients with the lowest SQUID scores (i.e., those patients with the lowest overall quality scores). They could also go a step further and identify the most complex patients (using the SQUID denominator) with low SQUID scores, to identify their more complex patients in need of improved care.

### Measuring SQUID reliability, responsiveness, validity, internal consistency, and distributional properties

Various statistical properties of the SQUID such as reliability, responsiveness, validity, and distributional properties were also of interest. Reliability refers to the degree to which two SQUID measures at different points close in time are correlated with one another, while responsiveness refers to whether the index detects clinically meaningful changes over time. To the extent that the patients' electronic medical record data is accurate, the measure is, by definition, essentially perfectly reliable. Responsiveness was investigated by examining the absolute increase in patients' and practices' SQUID values during this 15-month study period. Because the practices were participating in a QI project, it would be expected that patient-level and practice-level SQUIDs would increase significantly over time. Change over time was assessed for statistical significance using paired t-tests, linear regression, and the Wilcoxon sign test, as appropriate.

Validity refers to the degree to which the measure accurately reflects that which is being measured. Although several types of validity exist, we focused on face validity (i.e., a subjective assessment of whether the SQUID measures that which it was intended to measure). This property was assessed through an e-mail listserv for PPRNet members and through informal interviews with providers who participated in site visits or who attended the 2005 PPRNet A-TRIP network meeting in Seattle, WA.

Other statistical properties were also examined. It has also been recommended that performance measures based on multiple measures need to have good internal consistency, indicating that the individual items are measuring similar constructs [32]. Internal consistency was measured using Cronbach's alpha coefficient among the practices' third quarter 2005 scores on the individual quality indicators that comprise the SQUID. The intraclass correlation coefficient to determine the proportion of patient-level SQUID variation explained by practice membership was also calculated, by using a mixed linear regression model (SAS V9.1, Cary, NC), treating practice as a random effect. The distribution of E (the total number of eligible measures) was examined across the patient population to provide a general sense of its distribution, including the most frequent values observed and the associated variability. Lastly, histograms were created for patient- and practice-level SQUIDs from third quarter 2005 for use in determining their distributional properties, as this type of information may provide further insight into the overall nature of the variation in the quality of care provided. All analyses were performed with SAS 9.1 (Cary, NC).

## Results

**Figure 1:**
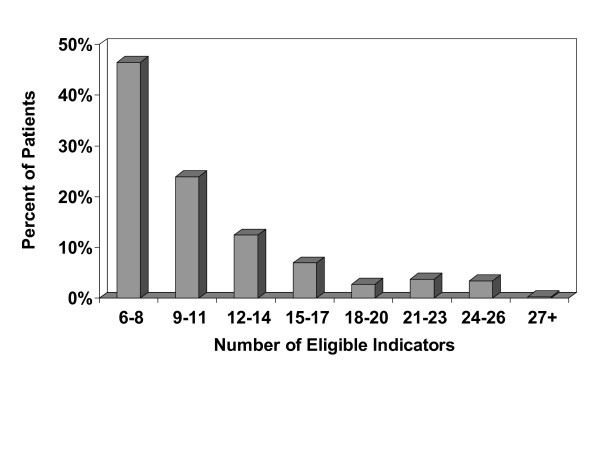
Histogram of third quarter 2005 patient-level total number of eligible indicators (n = 350,307 patients).

The third quarter 2005 population studied included 330,966 active adult patients in 89 active PPRNet primary care practices. Table 2 lists key descriptive statistics for these practices and patients within the practices. Most (78.7%) of the practices were family practices, with multiple providers. Of the diseases/conditions of interest, the most frequently reported were hypertension (24.6%) and hyperlipidemia (21.2%). A histogram reflecting the distribution of the total number of eligible indicators (E) is shown in Figure 1. Although E has a distribution that is skewed to the right, the way our indicators are defined, each adult has an E value that is 6 or greater. The median of E is 9, and the mean is 10.6 (s.d. = 4.9).

**Table 2:** Characteristics of 89 active A-TRIP practices as of September 30, 2005

Characteristic	Value
Specialty	
Internal Medicine n (%)	15 (16.9)
Family Medicine n (%)	70 (78.7)
Multi-Specialty n (%)	3 (3.4)
Gynecology: n (%)	1 (1.1)
Number of providers: mean (s.d.)	5.2 (6.2)
Number of active patients: mean (s.d.)	3,936 (4,308)
Age of active adult patients: mean (s.d.)	47.6 (17.8)
Gender of active adult patients: % male	40.6
Prevalence of selected morbidities reported among active adult patients	
Hypertension (%)	24.6
Hyperlipidemia (%)	21.2
Depression (%)	11.9
Diabetes mellitus (%)	8.7
Asthma (%)	5.3
Coronary heart disease (%)	3.3
COPD (%)	2.6
Heart failure (%)	1.4
Atrial fibrillation (%)	1.3
Alcohol abuse (%)	0.6

COPD: Chronic obstructive pulmonary disease

The responsiveness of patient and practice-level SQUIDs is highlighted in Table 3. Among patients who were active during the entire 15-month time period, the mean SQUID increased 3.6% (from 40.0% to 43.6%). Among all active patients (during the quarter of interest), the mean SQUID increased 3.2% (from 35.1% to 38.3%). Among practices that were active during the entire 15-month time period, the mean practice-level SQUID increased 3.8% (from 34.8% to 38.6%), with 88% of practices exhibiting a positive increase in their practice-level SQUID score. Additionally, analyses across the entire 3.5 year A-TRIP study indicated an adjusted average annual improvement in the SQUID of 2.43% (95% confidence interval 2.24% to 2.63%), an improvement that was consistent throughout the entire study. These changes were all significantly different from zero (p < 0.0001). The reason why the mean practice-level SQUIDs among patients active for the entire study are lower than the mean patient-level SQUIDs is due to patient turnover. The practice-level SQUIDs incorporate data from many patients who later became inactive during the time period, as well as new patients who join the practice. Since these two groups of patients did not have continual contact with their practice during the 15-month time period, their SQUID scores tended to be lower than the patients who were active throughout the study, thus reducing the values of the overall practice-level SQUIDs.

**Table 3:** Quarterly means, standard deviations (s.d), correlations among patient-level SQUIDs

Quarter	Mean^1 ^(s.d.) patient-level SQUID (n = 212,054)	Mean^2 ^(s.d.) patient-level SQUID [n]	Mean (s.d.) practice-level SQUID (n = 85)
Third quarter 2004	40.0% (20.1%)	35.1% (20.7%) [324,595]	34.8% (10.9%)
Fourth quarter 2004	41.3% (19.9%)	34.8% (20.8%) [355,381]	35.1% (10.6%)
First quarter 2005	42.5% (19.8%)	35.5% (21.0%) [360,682]	36.1% (10.5%)
Second quarter 2005	43.3% (19.7%)	36.4% (21.0%) [362,712]	37.0% (10.5%)
Third quarter 2005	43.6% (19.8%)	38.3% (21.0%) [330,966]	38.6% (10.6%)

^1 ^Mean SQUID among 212,054 patients active throughout entire study period

^2 ^Mean SQUID among patients active during the quarter of interest

When the SQUID algorithm and preliminary findings were presented to clinicians participating in site visits or attending the 2005 and 2006 PPRNet A-TRIP network meetings, feedback was favorable. During site visits, providers and staff reviewed practice-level SQUIDs to further assess their performance on A-TRIP measures. One practice used the trend of increasing SQUIDs to reinforce their focus on improving process measures related to preventive care (i.e., updating aspirin prescriptions in applicable patients, and sending letters to patients overdue for mammograms or colonoscopies). Another practice observed a decreasing trend in practice-level SQUIDs related to growth of their practice, and used their past performance as motivation for providing quality care to an influx of new patients. In general, providers appreciated the fact that the SQUID was an index that had a direct interpretation of the overall quality of care provided in their practices.

When PPRNet e-mail listerv members were asked to provide feedback on the SQUID, several interesting responses emerged, as they commented on how it was used in their practices. Direct quotes from this informal feedback request from physicians include:

"The SQUID ... provides an over-all indication of whether or not a practice is on a 'trajectory of improvement'. We find that there is 'psychic value' to knowing that."

"It's nice to have along with the [other] two graphs comparing us to the rest of the group. We just use it as an overall assessment of how we're doing."

" [We] have been using it as some information for my patients on how the practice does as a whole and for negotiations with insurers."

"We have used this extensively. I presented our data to the corporate fall conference. People were quite impressed. The insurance companies we work with also are excited about our improvements. We use the summary to give an overall view to ourselves (providers), the associates (staff), and others in our network. We follow this measure closely as a gauge of our progress. It would be interesting to use it for specific patients. We could have it to encourage compliance and congratulate successes for certain patients. I envision presenting a graph of that particular person's progress to him/her."

"Last year we had an influx of patients who work for [company X] and were being seen by other docs. Our summary indicator dipped and then came back up – the people at [company X] were most happy. It is a great lead-off slide for presentations...It is the future for medicine."

Patients' third quarter 2005 SQUIDs correlated relatively well (p < 0.0001) with their most recent systolic (r = -0.17) and diastolic (r = -0.23) BP (DM and HTN patients only), LDL (r = -0.26) (DM and CHD patients only), HDL (r = 0.17) (DM patients only), triglycerides (r = -0.16) (DM patients only), and A1C (r = -0.24) (DM patients only) measurements. The directionality of these associations also provide evidence of construct validity for the SQUID; that is, better overall quality was associated with lower values of BP, A1C, LDL, and triglyceride measures as well as higher values of HDL. The Cronbach's alpha coefficient among the practices' scores on the individual quality indicators was found to be 0.93, indicating excellent internal consistency. Although a low internal consistency would not necessarily be indicative of a poor composite measure, the fact that the SQUID does have a high Cronbach's alpha coefficient suggests that it is comprised of indicators measuring a common underlying quality construct.

**Figure 2:**
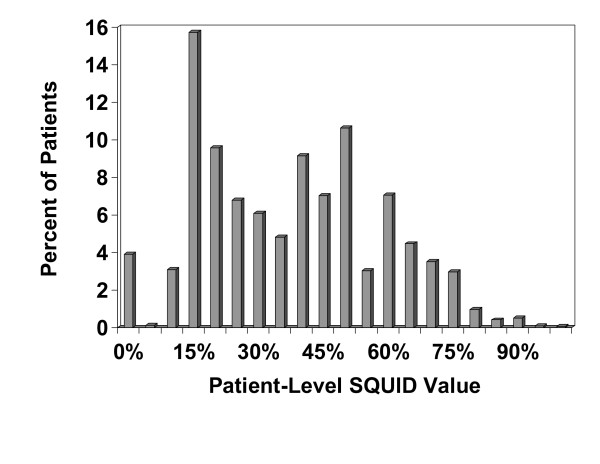
Histogram of third quarter 2005 patient-level SQUIDs (n = 350,307 patients).

**Figure 3:**
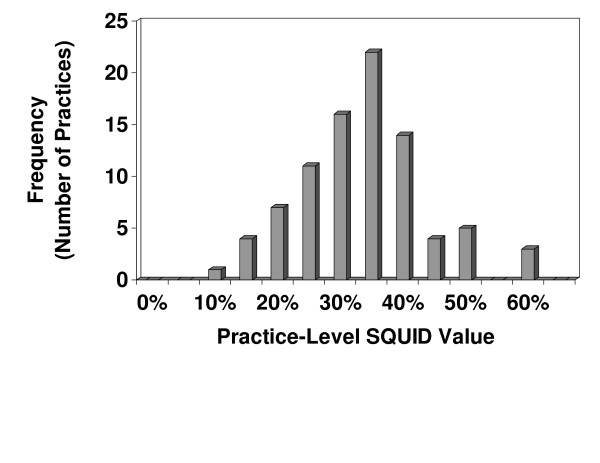
Histogram of third quarter 2005 practice-level SQUIDs (n = 89 practices).

A histogram of the third quarter 2005 patient-level SQUID values is shown in Figure 2. Note that approximately 4% of patients had SQUID values of zero, and the relatively bimodal distribution, with peaks between 15% and 20% and between 50% and 55%. A histogram of the third quarter 2005 practice-level SQUID values is shown in Figure 3. In contrast to the patient-level SQUIDs, the practice-level SQUID distribution was uni-modal. The average practice-level SQUID was 37.9%, with a standard deviation of 10.7%. The practice-level SQUIDs ranged from 12.3% to 68.3%, and the intra-class correlation coefficient, reflecting the proportion of SQUID variation explained by practice membership, was 23.8%.

## Discussion

This paper describes the Summary Quality Index (SQUID), a composite measure of healthcare quality in the primary care setting. The SQUID has several advantages compared with other composite quality measures. The algorithm is straightforward, and the resulting index satisfies the qualities of good performance measures and good outcome measures. Within the setting of A-TRIP, a QI demonstration project, it has been shown to be a reliable, responsive, and valid measure of healthcare quality. Feedback from clinicians suggests that this type of measure is quite appropriate and acceptable for primary care settings. They appreciate its use for tracking a summary measure of quality over time, and are excited about its potential for appealing internally to their clinical and clerical staff, as well as externally to insurers, corporate officials, and even their patients.

Having a patient-level composite measure is advantageous for several reasons, most notably it allows for comparisons across groups of patients with specific conditions (e.g., diabetes), demographics (e.g., the elderly), or types of care (e.g,. preventive or chronic) [33]. In fact, a subset of the A-TRIP quality indicators relevant to diabetes care has already been used in the development of the Diabetes-SQUID [30], which is ideal for studying ways to improve care for diabetes patients in the primary care setting. During the A-TRIP project, making the patient-level SQUIDs available to the clinicians responsible for the patients' care has allowed those clinicians to identify their most clinically complicated patients (i.e., based on the SQUID denominator values) along with their patients with the greatest need for care improvement (i.e., those with low SQUID scores). Using a composite measure may also be quite useful within QI projects involving multiple process and/or outcome measures within multiple clinical domains. Because efforts to improve quality in one area may yield declines in other areas, a summary measure may provide interested parties with a better sense of the resulting net increases or decreases in performance.

Because the SQUID can be calculated at the patient level, or aggregated to a higher level, such as that of the provider, practice, or health plan, it is useful from a variety of perspectives. As mentioned earlier, practices may use the patient-level SQUID to identify patients in most need of certain types of care. However, they may also use their practice-level SQUID as a marker of QI over time, or to compare their progress against that of other practices in their network. Health plans might use provider-level SQUIDs to rank providers or track progress over time, and researchers or QI organizations might use practice-level SQUIDs to rank practices or track them over time.

Because the denominator (referred to as 'E' in the algorithm) used in calculating the SQUID reflects the total number of relevant indicators for a given patient, a rather intuitive "complexity" adjustor is created in the process of calculating each patient's SQUID. Although this value (E) does not reflect the severity or duration of any individual patient conditions, it does reflect an overall level of complexity for that patient, because it includes a number of unique chronic conditions that are commonly treated in the primary care setting. This denominator can serve as a covariate in patient-level regression models for the purposes of complexity adjustment (analogous to risk adjustment), or it can be averaged across patients to serve as a complexity adjustor in provider or practice-level analyses.

This approach to quantifying overall quality of care is emerging as a useful tool in practice, in QI, and in research. Other algorithms mentioned in the literature for composite quality measures have typically been aimed at some aggregated level (rather than at the patient level), such as those used in physician or health plan profiling [4,5,9,10]. With the exception of the method described by CMS for quantifying multiple quality measures for hospitals [5], these algorithms involve the creation of some composite index that typically has no direct clinical interpretation. One set of methods that has been mentioned in the medical literature for combining multiple patient-level outcomes is the use of global statistical tests [11-14]. These tests can be an excellent way to account for correlated outcomes among patients in clinical trials; however, their effectiveness is limited when one or more of the outcomes is not relevant for significant numbers of patients (e.g., gender-specific measures such as whether a Pap test has been done in the past 3 years). The SQUID algorithm is similar to ones developed by CMS, RAND Corporation, and the VA [5,7,8]. The CMS methodology, however, has only been applied to the hospital setting, rather than at a patient or physician level, and likewise the VA aggregate indices are used as performance measures across groups of patients. The RAND methodology is broader in nature but relies on patient surveys and medical record abstracts.

There is much debate about the manner in which quality of healthcare should be measured [34]. For example, there are aspects of quality such as patient satisfaction, access to care, certain health outcomes, and efficiency that are not easily measured using electronic medical record or administrative data. Additionally, there is no consensus on whether quality should be measured as a single construct or as multiple domains [35]. Thus balancing what is practical and economical with what is desirable from various perspectives (e.g., patients, providers, insurers, and researchers) will likely continue to be a source of controversy.

The SQUID satisfies the criteria for a good outcome measure that can be used in clinical research studies, including being appropriate, reliable, responsive, precise, interpretable, acceptable, and feasible [36]. The SQUID also satisfies criteria for a desirable performance measure, as defined in a consensus document of the American Medical Association, the Joint Commission on Accreditation of Healthcare Organizations, and the National Committee for Quality Assurance [37]. These criteria included being of high priority for maximizing the health of persons or populations, financially important, able to demonstrate variation in care and/or the potential for improvement, based on established clinical recommendations, potentially actionable by users, and meaningful and interpretable to users. Another strength of this approach is that the SQUID can be easily adapted to reflect revisions in evidence for individual quality indicators.

The actual practice-level SQUID descriptive statistics (mean: 37.9%; standard deviation: 10.7%; range: 12.3% to 68.3%; intraclass correlation coefficient: 23.7%) may seem as a cause for concern, especially when compared to the RAND study's finding that adults in 12 metropolitan areas in the US received 54.9 percent of recommended care, ranging from 51% (Little Rock) to 59% (Seattle) [7]. However, the SQUID calculations for the PPRNet practices do rely on documentation of process of care within certain specific areas of the electronic medical record compared to patient telephone surveys and chart review by the RAND investigators. Thus we may have underestimated the true quality provided in these practices, due to some physicians opting to record data in the records in a manner (i.e., within the text of a progress note) that is not obtainable via the current PPRNet data extraction process.

The intraclass correlation coefficient for the patient-level SQUID (i.e., 23.8%) may seem relatively high in comparison with ICCs for outcomes of other studies [38,39]. However, because these practices were all involved to with a QI project during this time period, and since practices were allowed to determine the extent to which they participated in A-TRIP, we expected high variability in patient healthcare quality and that practice membership would explain much of this variation.

There are several limitations of this summary quality measure. Currently, each of the individual processes and outcomes comprising the SQUID is equally weighted, and it could be argued that certain process or outcome indicators should be weighted more heavily. Certain indicators may be viewed as being more clinically important than others, and other indicators may be easier to achieve than others. It is also possible that certain individual processes or outcomes may interact with one another, having synergistic or even antagonistic effects on overall quality; however, examining the influence of such interactions was beyond the scope of this study. Although it is possible that indicator-specific weights could be incorporated into the SQUID's summation formulas, deriving them would typically require building some type of group consensus or using statistical methodology such as factor analysis or item response theory methods [40]. One of the difficulties of these empirical approaches in the context of our patient population is the fact that many patients are not eligible for multiple measures; thus trying to determine how indicators cluster together or whether certain indicators are more difficult than others would require much more in-depth analyses that took into consideration eligibility differences among patients. Even if such analyses were conducted, resulting in a revised weighting scheme for each indicator, we would argue that such a process would result in a loss in the ease of interpretability of the SQUID, a factor we feel is key in communicating with an extremely varied audience that includes providers with varied levels of training and expertise (doctors, physician assistants, nurses), office staff, and even patients. Item weighting (or possibly item reduction) may, however, help address another potential limitation of the SQUID, that some of the individual indicators are correlated with one another. For example, practices that do well in measuring patients' total cholesterol routinely also tend to do well in measuring their patients' HDL and LDL cholesterol levels. Future research into possible weighting and/or item reduction schemes for the individual indicators could help sort out these issues. Additionally, as a general performance measure, the SQUID algorithm does not account for patient allergies or other contraindications to immunizations or medications; thus it would be virtually impossible for a practice to achieve a practice-level SQUID score of 100%. This fact is communicated to practices during site visits and network meetings, and practices are given a sense of what is practically achievable via internally derived SQUID benchmarks. One other potential limitation of the SQUID is the multi-modal nature of its distribution at the patient-level and the fact that it is bounded by 0% and 100%. Thus caution should be used when analyzing SQUID data from small numbers of patients. Lastly, although the SQUID may be useful in detecting general trends over time in quality, specific problematic areas within a given practice are likely more easily identified via individual- or condition-specific indicators.

## Conclusion

The SQUID has been a helpful tool in quantifying overall quality within the A-TRIP demonstration project. Providers have used the practice-level SQUIDs to assess overall performance on quality indicators in 8 clinical domains, and they have used the patient-level SQUIDs to identify the patients in most need of attention. A-TRIP research investigators have used it to identify practices making the largest gains in overall QI. The ability to identify these 'best practices' allows us to encourage dialogue between practices during annual A-TRIP network meetings, in which physicians, nurses, and other office staff share ideas to improve the quality of the care they provide. The SQUID values have also served as the primary outcomes in the final analyses of the A-TRIP project. Thus, it has benefit to patients, practitioners, insurers, and researchers.

## Competing interests

The author(s) declare that they have no competing interests.

## Authors' contributions

PJN participated in the SQUID methodology development, development of the feedback tools, statistical analysis, manuscript drafting, and critical review of the manuscript. AMW participated in the methodology development, manuscript drafting, and critical review of the manuscript. RGJ participated in the methodology development, development of the feedback tools, manuscript preparation, and critical review of the manuscript. CF participated in the methodology development, statistical analysis, and critical review of the manuscript. LN participated in the methodology development and critical review of the manuscript. SMO participated in the SQUID methodology development, development of the feedback tools, manuscript drafting, and critical review of the manuscript.
